# Modification of Heterotrimeric G-Proteins in Swiss 3T3 Cells Stimulated with *Pasteurella multocida* Toxin

**DOI:** 10.1371/journal.pone.0047188

**Published:** 2012-11-05

**Authors:** Rebecca C. Babb, Karen A. Homer, Jon Robbins, Alistair J. Lax

**Affiliations:** 1 King's College London, Department of Microbiology, Dental Institute, Guy's Hospital, London, United Kingdom; 2 King's College London, Wolfson Centre for Age-Related Disease, London, United Kingdom; Universidad de Costa Rica, Costa Rica

## Abstract

Many bacterial toxins covalently modify components of eukaryotic signalling pathways in a highly specific manner, and can be used as powerful tools to decipher the function of their molecular target(s). The *Pasteurella multocida* toxin (PMT) mediates its cellular effects through the activation of members of three of the four heterotrimeric G-protein families, G_q_, G_12_ and G_i_. PMT has been shown by others to lead to the deamidation of recombinant Gα_i_ at Gln-205 to inhibit its intrinsic GTPase activity. We have investigated modification of native Gα subunits mediated by PMT in Swiss 3T3 cells using 2-D gel electrophoresis and antibody detection. An acidic change in the isoelectric point was observed for the Gα subunit of the G_q_ and G_i_ families following PMT treatment of Swiss 3T3 cells, which is consistent with the deamidation of these Gα subunits. Surprisingly, PMT also induced a similar modification of Gα_11_, a member of the G_q_ family of G-proteins that is not activated by PMT. Furthermore, an alkaline change in the isoelectric point of Gα_13_ was observed following PMT treatment of cells, suggesting differential modification of this Gα subunit by PMT. G_s_ was not affected by PMT treatment. Prolonged treatment with PMT led to a reduction in membrane-associated Gα_i_, but not Gα_q_. We also show that PMT inhibits the GTPase activity of G_q_.

## Introduction

Heterotrimeric G-proteins are a family of key signal transduction proteins that intercede between the many G-protein coupled receptors (GPCR) that the cell uses to interrogate its local environment and downstream signalling pathways that ultimately regulate fundamental cellular choices [1]. G-proteins are divided into 4 classes (G_q_, G_12_, G_i_ and G_s_) according to their constituent alpha subunit, which is a guanine nucleotide binding protein that can exist in an inactive GDP-bound or an active GTP-bound form [2]. Activation of a GPCR causes a conformational change in its cognate Gα subunit that triggers GDP to be exchanged for GTP. The activated state persists until GTP is hydrolysed to GDP by the intrinsic GTPase activity of the Gα subunit. G-proteins are also subject to reversible tyrosine phosphorylation and lipid modifications during their activation cycle, but the regulatory role of these events is not fully understood [3]. Each G-protein class activates a characteristic set of downstream targets. The G_s_ and G_i_ families activate or inhibit adenylate cyclase, respectively [4]. The G_q_ family activates phospholipase C (PLC) [5], while the G_12_ family is particularly linked to activation of the Rho GTPase [6].

Intracellularly-acting bacterial protein toxins enzymatically modify a limited and precise set of cellular proteins to modulate their function. The *Pasteurella multocida* toxin (PMT) activates multiple signalling pathways in cultured cells leading characteristically to a strong mitogenic response [7]. PMT has been shown to activate members of the G_q_, G_12_ and G_i_ families [8]–[13]. PMT catalyses the deamidation of recombinant G_i_ at Gln-205 to inhibit its intrinsic GTPase activity [14]. We describe here the effects of PMT on all four classes of heterotrimeric G-proteins in Swiss 3T3 cells using two-dimensional (2-D) gel electrophoresis and other techniques.

## Materials and Methods

### Reagents

Cell culture reagents were obtained from Invitrogen. (γ-^32^P) GTP was obtained from PerkinElmer LAS. Anti-Gα_q/11_ (sc-392), anti-Gα_11_ (sc-394), anti-Gα_s_ (sc-387), anti-Gα_13_ (sc-410) and anti-Gα_i-2_ (internal: sc-7276) antibodies were from Santa Cruz Biotechnology. Anti-Gα_q_ (371752), anti-Gα_i-1_ (371720), anti-Gα_i-1-2_ (371723) and anti-Gα_i-1-3_ (371729: which is known to cross react with Gα_i-1_ and Gα_i-2_) antibodies were purchased from Calbiochem-Novabiochem. Phospho-FAK (Tyr^397^) was from New England Biolabs Ltd. All reagents used for 2-D gel electrophoresis were from GE HealthCare, unless otherwise stated. Recombinant PMT was purified essentially as described [15]. A recombinant His-tagged Gα_q_ subunit (371765) was purchased from Calbiochem-Novabiochem. Recombinant His6-tagged human Gα_i-1_ was expressed and purified from *E. coli* containing pProEX-HTb, which was provided as a kind gift by Professor David Siderovski (Department of Pharmacology, University of North Carolina, USA) [16]. All other chemical reagents were of analytical grade and were obtained from Sigma-Aldrich, unless otherwise stated.

### Cell culture

Swiss 3T3 cells, originally developed by Todaro and Green [17], and kindly provided by Theresa Higgins (Cancer Research UK, London, UK) were cultured as described [9]. Cells were grown to confluence and used when quiescent, before the addition of PMT or bombesin (Calbiochem-Novabiochem). The tyrosine kinase inhibitors Su6656 and St638 (Calbiochem-Novabiochem) were prepared in DMSO, diluted in DMEM containing 0.1% DMSO and added to cell cultures to give a final concentration of 100 µM 1 h prior to treatment with PMT.

### Preparation of Swiss 3T3 membranes and cytoplasmic fractions

Swiss 3T3 cells were grown in 145 mm dishes, rinsed twice with ice cold PBS and scraped into 2 ml of PBS containing proteinase inhibitors (Complete™, Roche Diagnostics). Cells from 10 dishes were pooled, collected by centrifugation (200 *g*, 10 min, 4°C), and washed cell pastes were frozen at −70°C until required. The frozen cell pastes (∼5 mg) were thawed on ice and suspended in 5 ml of membrane buffer (10 mM Tris-HCl, 10 mM MgCl_2_, 0.1 mM EDTA, pH 7.4, containing proteinase inhibitors). The cells were ruptured by 25 passes through a 23-gauge needle, and the resulting homogenate was centrifuged at 800 *g* for 10 min to remove unbroken cells and nuclei. The supernatants were transferred to fresh tubes and centrifuged at 50,000 *g* for 10 min. The supernatant containing cytoplasmic proteins was transferred to a fresh tube, snap frozen in liquid nitrogen and stored at −70°C. The pellet was washed and suspended in 10 ml of membrane buffer. After a second centrifugation step the membrane pellet was suspended in membrane buffer to a protein concentration of 1 mg/ml and stored at −70°C.

### SDS PAGE and urea gel electrophoresis

Membrane proteins were resolved by SDS PAGE on 12.8% acrylamide/0.06% bis acrylamide gels, or on these same gels containing 6M urea to separate the closely migrating Gα_11_ and Gα_q_ subunits as described [18]. Proteins were transferred to PVDF membranes and immunoblotted as described below.

### 2-D gel electrophoresis

Swiss 3T3 membrane proteins were resolved by 2-D gel electrophoresis, as described [19]. The immunodetection of Gα subunits was performed by incubating the membrane overnight at 4°C with primary antibody at a dilution of 1∶1000, followed by incubation with horseradish peroxidase-coupled secondary antibody at a dilution of 1∶10000 (SouthernBiotech) for 1 h at room temperature. The membrane was incubated with ECL™ chemiluminescent substrate (GE HealthCare) and signals were detected using an automatic X-Ray film processor (Jungwon Precision Industries Co.).

### Calcium microfluorimetry

Intracellular calcium was recorded as given previously [20]. Briefly, Swiss 3T3 cells were plated onto 19 mm glass cover slips and incubated in 5 µM Indo –AM (1 hour, 37°C, in the dark, Calbiochem). Cover slips were placed in a custom built chamber allowing gravity fed superfusion (10–12 ml/min) of a modified Krebs solution. Bombesin was applied by switching a multiway tap to a solution containing it and was removed by switching back to a bombesin free solution. The waste was removed by a peristaltic pump. Recordings were performed at room temperature by subtraction of background light and recording the emitted light from individual cells at 405 and 488 nm. The emission ratio (R) was converted to a calcium concentration after calibration (see reference 20] in which [Ca]i (nM) = 1028(R-0.86)/(12-R) and autofluorescence was less that 4%. .

### Trypsin protection assay

The trypsin protection assay was adapted from Evanko *et al.* [21]. Briefly, membrane fractions (100 µg) were incubated with PMT, bombesin, GTPγS or GTP at the required concentrations at 37°C for times indicated. Membrane fractions were centrifuged at 18,000×g for 10 min at 4°C and the pellet was resuspended in 12.8 µl of solubilisation buffer (20 mM Tris-HCl, pH 7.5, 100 mM NaCl, 2 mM MgCl_2_, 0.1 mM EDTA, 1 mM dithiothreitol, 10% glycerol, 1% C_12_E_10_ (polyoxyethylene 10-lauryl ether), 0.1 mM phenylmethylsulfonyl fluoride), vortexed, incubated on ice for 20 min and centrifuged at 18,000×g for 10 min at 4°C. The supernatant was then transferred to a new microfuge tube, treated with 4 µl of trypsin mixture (100 µM GDP, 1.5 mg/ml trypsin in solubilisation buffer) for 30 min at 30°C. The trypsin activity was neutralised with 3 µl of soybean trypsin inhibitor (3 mg/ml). Trypsin-resistant fragments were resolved by SDS-PAGE, and detected by immunoblotting using antiserum against Gα_q/11_. The induction of trypsin protection by GTPγS and GTP alone or in the presence of bombesin or PMT were quantified relative to untrypsinised G_q_ using scanning densitometry (GeneTools, Syngene). Data were analysed using factorial analysis of variance (ANOVA) by Dr Ron Wilson (King's College London). Unactivated Gα subunits (GDP-bound) are highly susceptible to tryptic digestion; however tryptic cleavage is inhibited when G-proteins are activated (GTP-bound) as most cleavage sites are conformationally protected, and a product resulting from a small N-terminal cleavage can be visualised [22].

### Measurement of high-affinity GTPase activity

Determination of GTPase activity was essentially as described [23]. High-affinity GTPase activity was determined by subtraction of P_i_ release in membranes incubated with 50 µM of GTP (low-affinity GTPase activity) from that with 0.5 µM GTP (total GTPase activity).

## Results

### PMT stimulates an acidic modification of Gα_q_ and Gα_i_ family proteins

**Figure 1:**
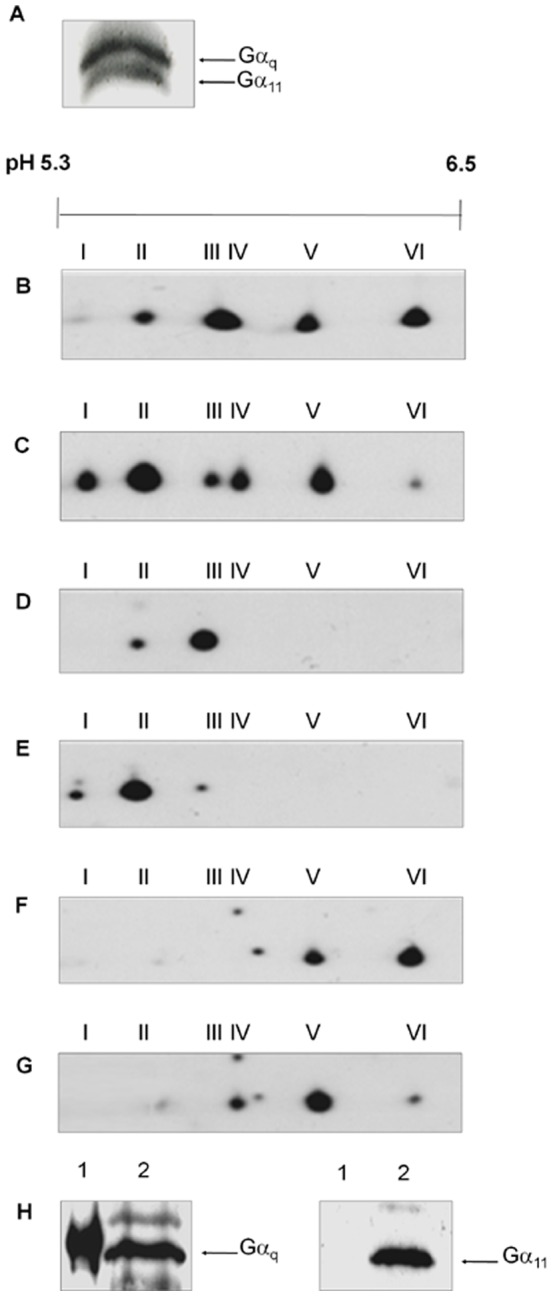
PMT induces the covalent modification of Gα_q_ and Gα_11_. (**A**) Membrane proteins from Swiss 3T3 cells were separated by urea gel electrophoresis and Western blotted with anti-Gα_q/11_ antibody. The locations of Gα_q_ and Gα_11_ are indicated by arrows. Membrane proteins from Swiss 3T3 cells (**B**, **D**, **F**) left untreated or (**C**, **E**, **G**) treated with 150 pM PMT for 4 h were separated by 2-D gel electrophoresis and Western blotted with (**B**, **C**)anti-Gα_q/11_, (**D**, **E**)anti-Gα_q_ or (**F**, **G**) anti-Gα_11_ antibody. (**H**) Recombinant Gα_q_ subunit (lane 1) and membrane proteins from Swiss 3T3 cells (lane 2) were separated by SDS PAGE and Western blotted with anti-Gα_q_ (left panel) or -Gα_11_ (right panel) antibody. Samples from at least 3 independent experiments were resolved with similar results.

Gα_q/11_ antiserum detected both Gα_q_ and Gα_11_ subunits at an apparent molecular mass of 42 kDa in membranes prepared from quiescent Swiss 3T3 cells. Separating these subunits on a urea gel showed that Gα_q_ (which aberrantly runs slower in this system than G_11_ [24]) was more abundantly expressed than Gα_11_ in these cells (Fig. 1A). A similar relative abundance has been shown in rat neurons [25]. Four distinct Gα_q/11_ molecular isoforms, designated q-II, q-III, q-V and q-VI, were resolved by 2-D gel electrophoresis followed by immunoblotting with anti-G_q/11_ antibody in membranes derived from untreated cells (Fig. 1B). These plus two additional isoforms, q-I and q-IV, were detected by 2D PAGE and Western blot analysis of membrane fractions derived from cells treated with PMT (150 pM) for 4 h (Fig. 1C; Table 1).

**Table 1:** Analysis of pI values of Gα_q_ family isoforms after treatment with PMT.

	Control (pI)			PMT-treated (pI)		
Isoform	Gα_q/11_	Gα_q_	Gα_11_	Gα_q/11_	Gα_q_	Gα_11_
**q-I**	-	-	-	5.39±0.04	5.42±0.02	-
**q-II**	5.45±0.04	5.49±0.02	-	5.49±0.01	5.51±0.02	-
**q-III**	5.61±0.05	5.59±0.05	-	5.60±0.08	5.60±0.03	-
**q-IV**	-	-	-	5.64±0.01	-	5.64±0.02
**q-V**	5.76±0.09	-	5.75±0.01	5.76±0.09	-	5.73±0.01
**q-VI**	5.89±0.1	-	5.85±0.01	5.83±0.02	-	5.82±0.05

The samples were as described in the legend to Figure 1 and the results are expressed as the mean ± standard error of the mean (n = 3).

Antiserum directed only against Gα_q_ detected two isoforms with pI values corresponding to q-II and q-III (Fig. 1D; Table 1) in untreated cells. The Gα_q_ antiserum detected an additional isoform with a pI value corresponding to q-I in PMT-treated cells (Fig. 1E; Table 1). Antiserum directed only against Gα_11_ detected two isoforms in untreated cells with pI values corresponding to the isoforms q-V and q-VI (Fig. 1F; Table 1) and one additional isoform with a pI value corresponding to q-IV in PMT-treated cells (Fig. 1G; Table 1).

We excluded the possibility that the Gα_11_ antibody could react with Gα_q_ by testing the ability of the Gα_q_ and Gα_11_ antibodies to react with a recombinant Gα_q_ subunit. The Gα_q_ but not the Gα_11_ antiserum could detect the Gα_q_ subunit (Fig. 1H). The experimentally determined pI values for the Gα_q/11_ isoforms (Table 1) are similar to the predicted pI values of 5.48 and 5.70 for murine Gα_q_ and Gα_11_, respectively [26], [27].

**Figure 2:**
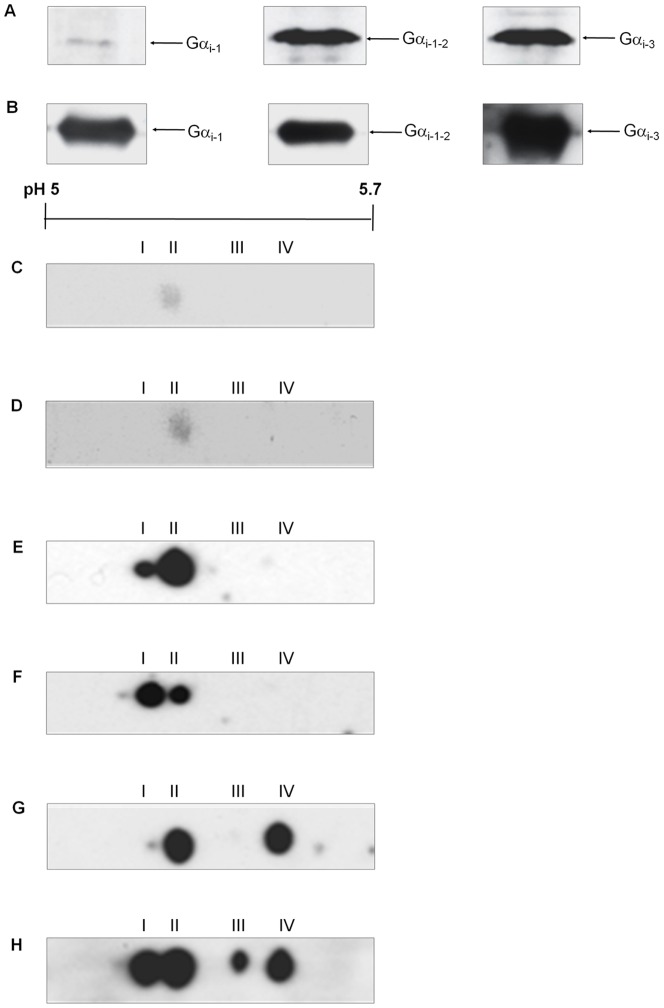
PMT induces the covalent modification of Gα_i_. (**A**) Membrane proteins from Swiss 3T3 cells were separated by SDS PAGE and Western blotted with anti-Gα_i-1_, anti-Gα_i-1-2_ or anti-Gα_i-1-3_ antibody, as indicated. (**B**) A recombinant Gα_i-1_ subunit was analysed by SDS PAGE and Western blotted with anti-Gα_i-1_, -Gα_i-1-2_ or -Gα_i-1-3_ antibody, as indicated. Membrane proteins from Swiss 3T3 cells (**C**, **E**, **G**) left untreated or (**D**, **F**, **H**) treated with 150 pM PMT for 4 h were separated by 2-D gel electrophoresis and Western blotted with (**C**, **D**) anti-Gα_i-1_, (**E**, **F**)anti-Gα_i-1-2_ or(**G**, **H**) anti-Gα_i-1-3_ antibody. Samples from at least 3 independent experiments were resolved with similar results.

The expression of the Gα_i-1_, Gα_i-2_ and Gα_i-3_ subclasses, which have the widest tissue expression pattern of this family [28], was analysed in Swiss 3T3 cells using specific antisera. The Gα_i-1-2_ (directed against Gα_i-1_ and Gα_i-2_) and Gα_i-1-3_ antisera (directed against Gα_i-1_, Gα_i-2_ and Gα_i-3_) each detected an abundant protein band at 40 kDa in membranes from Swiss 3T3 cells. The antiserum specific for only Gα_i-1_ detected a weak band (Fig. 2A), although this antiserum could be shown to react strongly with a recombinant Gα_i-1_ subunit (Fig. 2B), demonstrating a low abundance of Gα_i-1_ in Swiss 3T3 cells.

Gα_i-1_ isoforms were present at low abundance in membranes prepared from either untreated or PMT-treated Swiss 3T3 cells as determined by 2-D gel electrophoresis followed by immunoblotting (Fig. 2C, D; Table 2). The Gα_i-1-2_ antiserum detected two Gα_i_ isoforms in untreated and PMT-treated cells, designated i-I and i-II (Fig. 2E, F; Table 2), with a reproducible change in the relative abundance of the isoforms after PMT treatment. The Gα_i-1-3_ antiserum detected 3 Gα_i_ isoforms in untreated cells, two of which appeared to correspond to i-I and i-II; the third isoform was designated i-IV (Fig. 2G; Table 2). The Gα_i-1-3_ antiserum also detected these and one additional isoform, i-III in PMT-treated cells (Fig. 2H; Table 2).

**Table 2:** Analysis of pI values of Gα_i_ family isoforms after treatment with PMT.

	Control (pI)			PMT-treated (pI)		
Isoform	Gα_i-1_	Gα_i-1,2_	Gα_i-1,2,3_	Gα_i-1_	Gα_i-1,2_	Gα_i-1,2,3_
**i-I**	-	5.11±0.01	5.09±0.03	-	5.10±0.01	5.07±0.02
**i-II**	-	5.22±0.03	5.18±0.03	-	5.17±0.01	5.17±0.02
**i-III**	-	-	-	-	-	5.34±0.02
**i-IV**	-	-	5.59±0.03	-	-	5.45±0.01

The samples were as described in the legend to Figure 2 and the results are expressed as the mean ± standard error of the mean (n = 3).

The predicted pI values of murine Gα_i-1_, Gα_i-2_ and Gα_i-3_ are 5.69, 5.28 and 5.50, respectively [29]. It seems probable that isoforms i-I and i-II detected by the Gα_i-1-2_ antiserum belong to the Gα_i-2_ subclass, as isoforms of the Gα_i-1_ subclass are expected to have a more basic pI, and Gα_i-1_ was not detected in Swiss 3T3 cells. Isoforms i-III and i-IV are therefore likely to belong to the Gα_i-3_ subclass. Orth *et al.* resolved Gα_i-1_ and Gα_i-2_ from mouse embryonic fibroblast cells by 2-D gel electrophoresis at an unspecified pI value and showed that PMT treatment of these cells caused an acidic pI shift consistent with deamidated recombinant Gα_i-2_ [14]. Our results suggest that PMT catalyses the acidic covalent modification of Gα_i-2_ and Gα_i-3_.

### PMT induces an alkaline modification of Gα_13_

**Figure 3:**
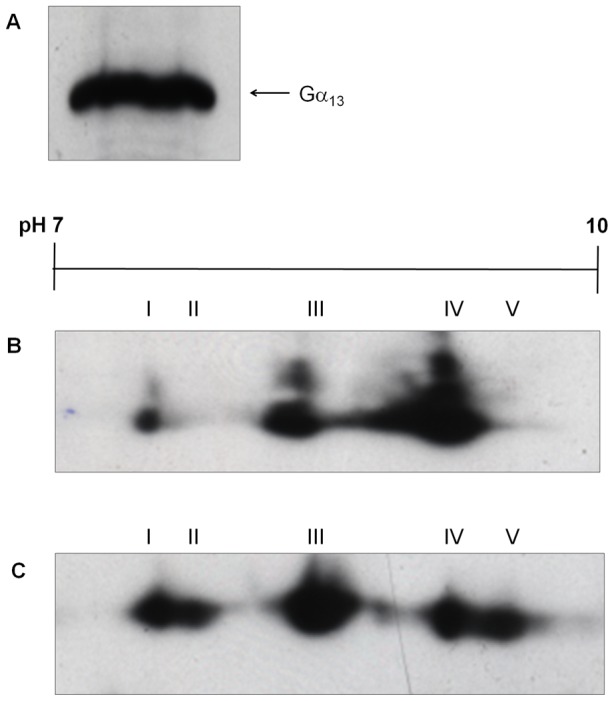
PMT induces the covalent modification of Gα_13_. (**A**) Membrane proteins from Swiss 3T3 cells were separated by SDS PAGE and Western blotted with anti-Gα_13_ antibody. The location of Gα_13_ is indicated. Membrane proteins from Swiss 3T3 cells left (**B**) untreated or (**C**) treated with 150 pM PMT for 4 h were separated by 2-D gel electrophoresis and Western blotted with anti-Gα_13_ antibody. Samples from at least 3 independent experiments were resolved with similar results.

The two members of the Gα_12_ family, Gα_12_ and Gα_13_, are ubiquitously expressed [30]. Gα_13_ was detected in Swiss 3T3 membranes using antiserum against Gα_13_ (Fig. 3A). Three Gα_13_ isoforms, 13-I, 13-III and 13-IV, were identified in membranes from Swiss 3T3 cells (Fig. 3B). Two additional isoforms, 13-II and 13-V, were detected in membranes derived from PMT-treated cells (Fig. 3C; Table 3). The additional Gα_13_ isoforms seem to be the result of an alkaline pH shift, in contrast to the effect of PMT on Gα_q/11_ and Gα_i_ isoforms. Under our experimental conditions, Gα_12_ could not be resolved by 2-D gel electrophoresis.

**Table 3:** Analysis of pI values of Gα_13_ isoforms after treatment with PMT.

	Control (pI)	PMT-treated (pI)
Isoform	Gα_13_	Gα_13_
**13-I**	8.15±0.02	8.15±0.07
**13-II**	-	8.24±0
**13-III**	8.54±0.02	8.54±0.02
**13-IV**	8.90±0.05	8.90±0.01
**13-V**	-	9.04±0.01

**Figure 4:**
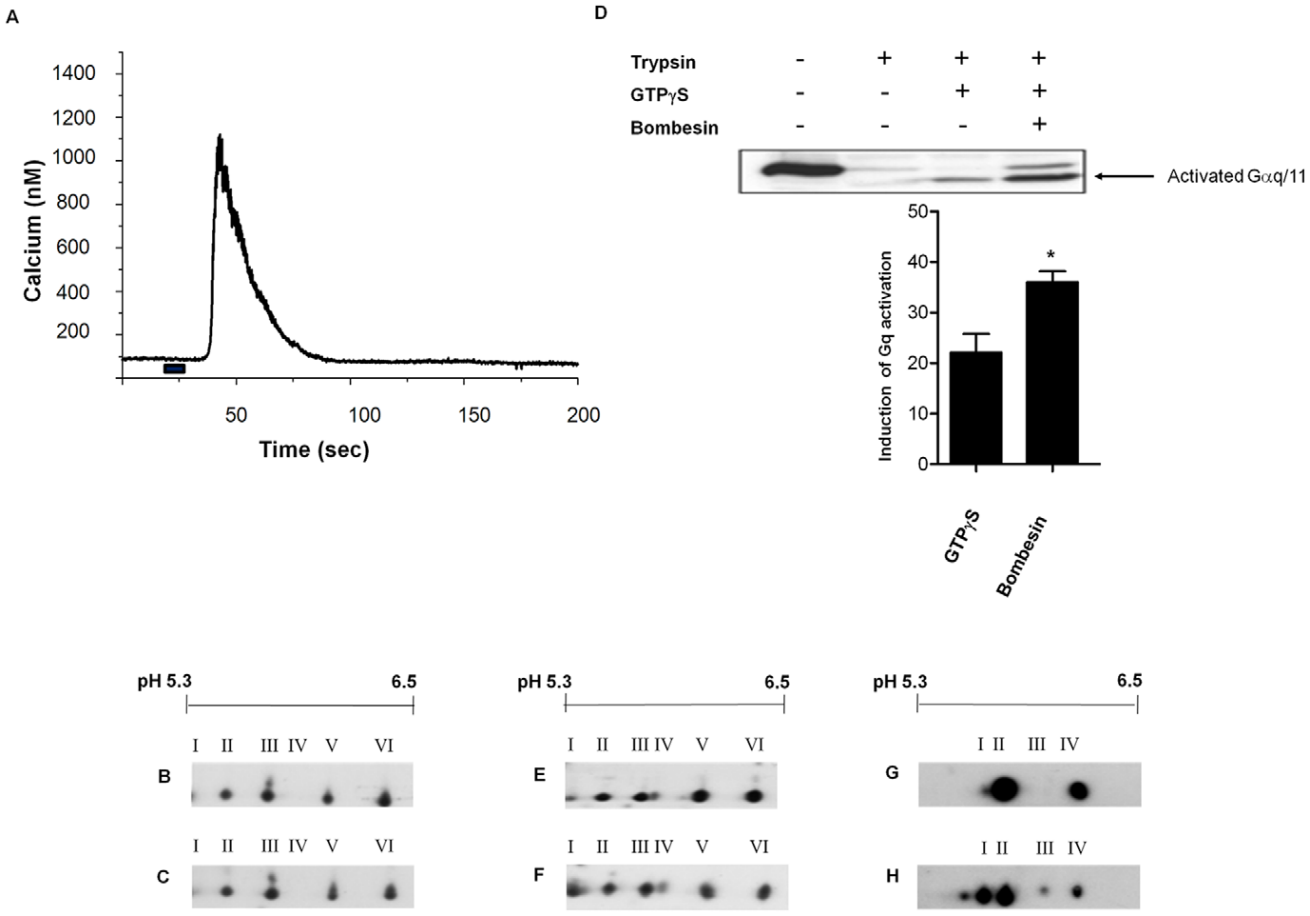
Sodium vanadate treatment mimics PMT effects on Gα_q_ and Gα_i_. (**A**) Indo-1 AM labelled Swiss 3T3 cells were treated with 30 nM bombesin for 10 s (marked with solid bar beneath trace) and intracellular Ca^2+^ release was measured. Membrane proteins from Swiss 3T3 cells that were either (**B**) untreated or (**C**) treated with 30 nM bombesin for 1 min were separated by 2-D gel electrophoresis and Western blotted with anti-Gα_q/11_ antibody. (**D**) Swiss 3T3 membrane proteins were incubated in the presence or absence of 30 nM bombesin for 20 min with or without 0.05 nM GTPγS. The proteins were then analysed for trypsin protection as described under Materials and Methods, and activated Gα_q/11_ was separated by SDS PAGE and Western blotted with anti-Gα_q/11_ antibody. Quantification of activated Gα_q/11_ (lower panel) was determined by densitometric scanning and these data were analysed using factorial analysis of variance (ANOVA). The induction of activation shown is relative to the density of the band without GTPγS or bombesin. Bombesin significantly enhanced GTPγS binding to Gα_q/11_ (* p = 0.002). Membrane proteins from Swiss 3T3 cells were incubated with (**E**) 1 mM sodium vanadate for 20 min at 37°C or (**F**) 1 mM sodium vanadate and 30 nM bombesin for 20 min at 37°C, proteins were separated by 2-D gel electrophoresis and Western blotted with anti-Gα_q/11_ antibody. Membrane proteins from Swiss 3T3 cells were incubated (**G**) without or (**H**) with 1 mM sodium vanadate for 20 min at 37°C, proteins were separated by 2-D gel electrophoresis and Western blotted with anti-Gα_i-1-3_ antibody. Samples from at least 3 independent membrane preparations were resolved with similar results.

The samples were as described in the legend to Figure 4 and the results are expressed as the mean ± standard error of the mean (n = 3).

### PMT does not induce any modification of Gα_s_

The alpha subunits of the ubiquitously expressed G_s_ family can be expressed as four distinct forms as a result of alternative mRNA splicing [31]. Swiss 3T3 cells were shown to express both large (55 kDa) and small (52 kDa) forms of Gα_s_, with Gα_s_-large being more abundantly expressed than Gα_s_-small (Fig. S1A). Six isoforms of Gα_s_-large (s-I to s-VI) and two isoforms of Gα_s_-small (s-VII and s-VIII) were resolved in membranes derived from Swiss 3T3 cells by 2-D gel electrophoresis, followed by immunoblotting with the Gα_s/olf_ antiserum (Fig. S1B; Table S1). The Gα_s_-large isoforms were detected at a more acidic pI than the Gα_s_-small isoforms, which concurs with previous findings [19]. PMT showed no discernable effect on the pI or molecular mass of the Gα_s_ subunits (Fig. S1C; Table S1).

### PMT stimulates the stable covalent modification of G-proteins

It was important to establish whether the additional isoforms detected in PMT-treated cells arose as a consequence of normal activation induced by PMT or if they were directly PMT-modified. Cells were challenged with the neuropeptide bombesin, which acts through a G_q_-coupled receptor to stimulate phospholipase C (PLC) activation culminating in the release of Ca^2+^ from intracellular stores [32]. Bombesin at a concentration of 30 nM effectively stimulated Ca^2+^ release from cells (Fig. 4A), but no additional Gα_q/11_ isoforms were detected by 2D PAGE and Western blot analysis of a membrane fraction derived from cells exposed to bombesin (Fig. 4B, C). This suggested that Gα_q_-coupled receptor activation did not stimulate the stable covalent modification of Gα_q_.

We have previously demonstrated that PMT induced the phosphorylation of Gα_q_ on Tyr369 [9]. We stimulated membrane fractions with bombesin in the presence of sodium vanadate, a potent tyrosine phosphatase inhibitor, in order to prevent the dephosphorylation of Gα_q/11_. Bombesin activation of Gα_q/11_ in the membrane fractions was confirmed by the trypsin protection assay. Bombesin significantly enhanced GTPγS binding to Gα_q/11_ (p = 0.002), by up to 50% in some cases, the most likely explanation being that its action accelerated the rate of nucleotide exchange (Fig. 4D). The additional isoforms detected in membranes stimulated with bombesin appeared to be identical to those found in PMT-treated cells. However, the additional Gα_q/11_ and Gα_i_ isoforms were also found in membranes derived from unstimulated cells, that had been treated with sodium vanadate alone (Fig. 4E, F and H). These findings suggest that PMT modification of Gα_q/11_ and Gα_i_ produces a similar pI shift as the tyrosine phosphorylation of these Gα subunits.

The appearance of the additional isoforms observed in PMT-treated cells could not be blocked by the competitive kinase inhibitors Su6656 or St638, although these inhibitors were effective at blocking pervanadate-induced phosphorylation of focal adhesion kinase (FAK) (Fig. S2). We have previously shown that a mutant PMT (PMT^C1165S^) can stimulate the tyrosine phosphorylation of G_q_, although it does not activate G_q_ downstream signalling [9]. Treatment of Swiss 3T3 cells with PMT^C1165S^ did not result in the covalent modification of Gα_q_ or Gα_i_ (Fig. S3). Moreover, tyrosine phosphorylation is a transient reversible modification that cannot be readily detected unless tyrosine phosphatases are inhibited.. The PMT-induced modification of Gα subunits was detected in the absence of sodium vanadate, indicating that the PMT-induced modification was covalent and stable.

### Prolonged treatment of cells with PMT has differential effects on G-proteins

**Figure 5:**
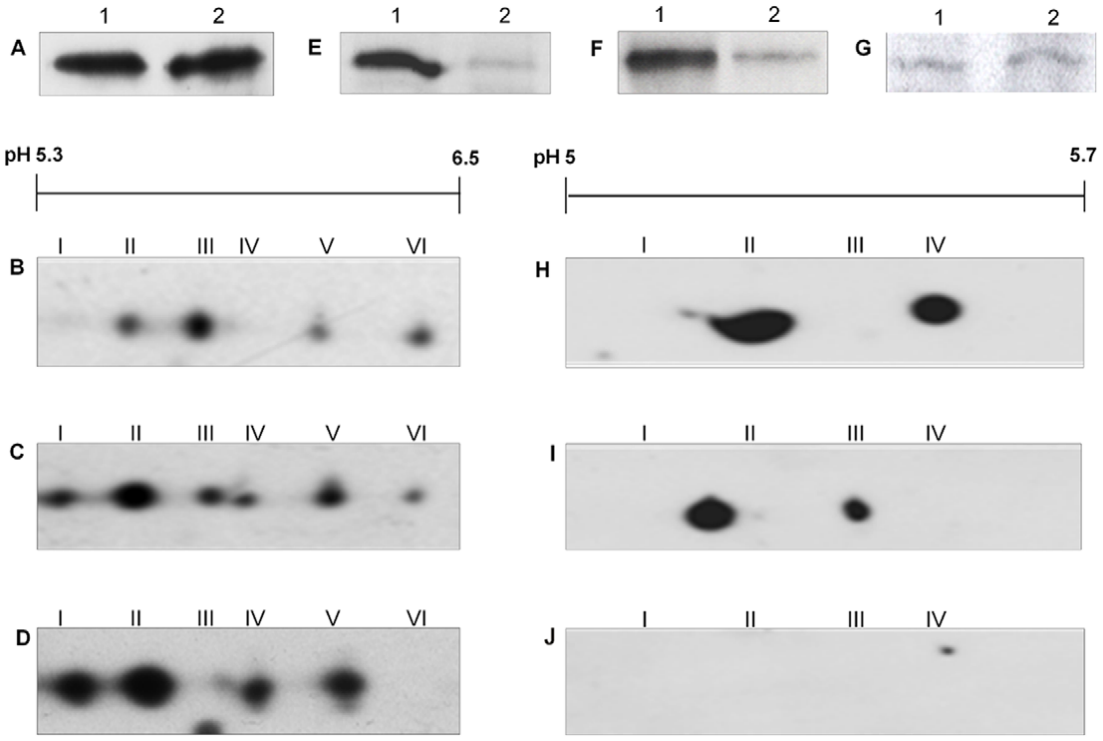
Prolonged exposure of Swiss 3T3 cells to PMT causes the loss of Gα_i_ but not G_q_ from cell membranes. (**A**) Membrane proteins from Swiss 3T3 cells left untreated (lane 1) or treated with PMT at 1 nM for 16 h (lane 2) were separated by SDS PAGE and Western blotted with anti-Gα_q/11_ antibody. Membrane proteins from Swiss 3T3 cells (**B**) left untreated, or treated with 1 nM PMT for (**C**) 4 h or (**D**) 16 h were separated by 2-D gel electrophoresis and Western blotted with anti-Gα_q/11_ antibody. Samples from at least 3 independent experiments were resolved with similar results. Membrane proteins from Swiss 3T3 cells left untreated (lane 1) or treated with 1 nM PMT for 16 h (lane 2) were separated by SDS PAGE and Western blotted with (**E**) anti-Gα_i-1-3_ antibody or (**F**) an antibody recognising an internal epitope of Gα_i-2_. (**G**) Cytoplasmic proteins from Swiss 3T3 cells left untreated (lane 1) or treated with 1 nM PMT for 16 h (lane 2) were separated by SDS PAGE and Western blotted with anti-Gα_i-1-3_ antibody. Membrane proteins from Swiss 3T3 cells (**H**) left untreated, treated with 1 nM PMT for (**I**) 4 h or (**J**) 16 h were separated by 2-D PAGE and Western blotted with anti-Gα_i-1-3_ antibody. Samples from 3 independent experiments were resolved with similar results.

PMT treatment decreased the abundance of some of the pre-existing Gα_q/11_ and Gα_i_ isoforms in membrane fractions. To explore if PMT caused G-protein removal from membranes, Swiss 3T3 cells were treated with PMT at a concentration of 1 nM for 16 h. This treatment did not cause loss of Gα_q/11_ from the membrane (Fig. 5A), but resulted in the complete loss of the most basic isoforms of Gα_q_ and Gα_11_, q-III and q-VI, respectively (Fig. 5B, C, and D), while isoforms q-II and q-IV did not undergo an evident change in abundance. We speculate that the loss of detection of Gα_q/11_ isoforms q-III and q-VI is a result of the covalent modification of these isoforms to q-I and q-IV, respectively, induced by PMT.

In contrast, prolonged treatment of Swiss 3T3 cells with PMT generally resulted in the almost complete loss of Gα_i_ from membranes (Fig. 5E, F). It is unlikely that the failure to detect the Gα_i_ isoforms reflects a modification that interferes with the Gα_i-1-3_ antigen recognition site, which is at the C-terminus of Gα_i_, as the loss of Gα_i_ from membranes could also be demonstrated with an antiserum against an internal epitope of Gα_i-2_ (Fig. 5F). Cytoplasmic extracts of cells that had received prolonged treatment with PMT were probed with anti-Gα_i-1-3_ antibody, but no increase in Gα_i_ subunits could be detected in these fractions (Fig. 5G). It appears that the sequential loss of Gα_i_ from membranes proceeds by covalent modification of Gα_i_ isoforms i-II and i-IV to produce isoforms i-I and i-III, respectively, followed over time by the loss of isoforms i-I and i-III from the membranes (Fig. 5H–J). In some cases only partial loss of Gα_i_ isoforms was observed over this time period (data not shown).

### PMT inhibits the GTPase activity of G_q_

**Figure 6:**
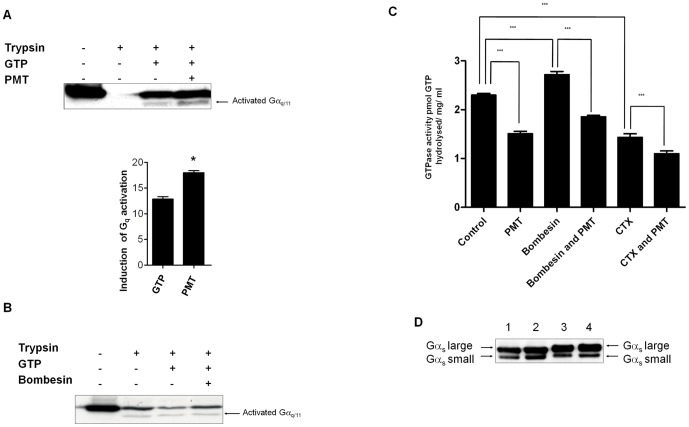
PMT inhibits the GTPase activity of G_q_. (**A**) Membrane proteins were incubated in the presence or absence of 150 pM PMT for 1 h with 0.5 nM GTP and tested in a trypsin protection assay as described in Materials and Methods. Proteins were separated by SDS PAGE and Western blotted with anti-Gα_q/11_ antibody. Quantification of activated Gα_q/11_ (lower panel) was determined by densitometric scanning and the data were analysed using factorial analysis of variance (ANOVA). The induction of activation shown is relative to the density of the band without GTP or PMT. PMT significantly enhanced GTP binding to G_q_ (* p = 0.03). (**B**) Membrane proteins were incubated in the presence or absence of 30 nM bombesin 20 min with 0.5 nM GTP and tested in a trypsin protection assay as described in Materials and Methods. Proteins were separated by SDS PAGE and Western blotted with anti-Gα_q/11_ antibody. Samples from at least 3 independent membrane preparations were resolved with similar results. (**C**) Membranes derived from Swiss 3T3 cells that had either been treated or untreated with 150 pM PMT for 4 h or 100 ng cholera toxin, or both, were treated with or without 30 nM bombesin for 20 min in the presence of [γ-^32^P] GTP. All the experimental conditions were repeated three times, and all data are presented as mean ± standard deviation (SEM). The results for the groups were compared using single-factor analysis of variance (one-way ANOVA), followed by Newnan-Keuls test used to determine differences between groups. Significant changes are indicated by an asterisk (* *P*<0.05, *** *P*<0.001). (**D**) Membranes derived from Swiss 3T3 cells that had either been untreated (lane 1) or treated with 150 pM PMT for 4 h (lane 2), or 100 ng cholera toxin for 16 h (lane 3), or both PMT and cholera toxin (lane 4) were resolved by SDS PAGE followed by Western blotting with an anti-Gα_s_ antibody. Samples from at least 3 independent membrane preparations were resolved with similar results.

PMT did not significantly enhance GTPγS binding to Gα_q/11_ in contrast to bombesin (data not shown). Due to its enzymatic nature, PMT required a longer incubation time to promote GTP binding to Gα_q_ compared to bombesin [9]. Therefore, it is likely that during the course of the incubation, Gα_q_ was gradually saturated by GTPγS, thereby preventing the detection of PMT-enhanced GTPγS binding to Gα_q_ above background levels. When GTP was used instead of GTPγS, PMT significantly enhanced GTP binding to Gα_q_ as measured by trypsin protection (p = 0.03), by up to 30% (Fig. 6A), in contrast to bombesin (Fig. 6B). This finding suggested that PMT might inhibit the GTPase activity of Gα_q_, to prevent the hydrolysis of GTP to GDP.

Bombesin stimulated the steady-state GTPase activity in Swiss 3T3 membrane preparations by up to 30%, whereas pre-treatment of cells with PMT at 150 pM for 4 h reduced the basal and bombesin-stimulated GTPase activity in membrane preparations (Fig. 6C). To further decrease the basal steady-state GTPase level, cells were pre-treated with cholera toxin, which ADP-ribosylates Gα_s_ to inhibit its GTPase activity. Cholera toxin caused an increase in the molecular weight of both the long and short forms of Gα_s_, due to the addition of ADP ribose (Fig. 6D). Pre-treatment of cells with both cholera toxin and PMT further decreased the basal GTPase activity in membrane preparations, compared to cells pre-treated with PMT alone. Bombesin stimulated the steady state GTPase activity by up to 50% in cells pre-treated with cholera toxin, whereas the additional pre-treatment of cells with PMT reduced the bombesin-stimulated GTPase activity in membrane preparations, indicating that PMT inhibits the GTPase activity of Gα_q_ but not G_s_ (Fig. 6C).

## Discussion

PMT executes its cellular effects through the activation of the heterotrimeric G-proteins, G_q_, G_12_ and G_i_ [8]–[13]. This has been shown to occur in recombinant G_i_ by PMT-induced deamidation of Gln-205 to glutamic acid, which inhibits its intrinsic GTPase activity [14]. The work we report here complements these studies by investigating covalent modifications of G-proteins in Swiss 3T3 cells treated with PMT. PMT treatment consistently led to the appearance of new isoforms at a lower pI for both Gα_q_ and Gα_11_. PMT also stimulated the covalent modification of members of the G_i_ family. The Gα_12_ family proteins, unlike the other G-protein families, have predicted pI values within the alkaline pH range (>pH 8) and such proteins are difficult to resolve by 2-D gel electrophoresis [33]. Gα_13_, but not Gα_12_, subunits displayed a reproducible pattern and PMT treatment led to new Gα_13_ isoforms at slightly higher pI values. We found no evidence that PMT stimulates the covalent modification of Gα_s_, although the glutamine residue targeted by PMT is conserved in all G-proteins.

Stimulation of G_q_-coupled receptors by bombesin only resulted in the detection of the additional Gα_q/11_ isoforms observed in PMT-treated cells when vanadate was present. The addition of sodium vanadate per se led to a similar pattern of isoforms to those observed in PMT-treated cells. However it is likely that these different treatments lead to different modifications. The modification of Gα_q/11_ and Gα_i_ stimulated by PMT was detected without sodium vanadate, and is thus indicative of a stable covalent modification such as deamidation, whereas tyrosine phosphorylation is a transient covalent modification. We previously showed that a src family kinase mediates the phosphorylation of G_q_ in response to PMT [9]. However, pre-treatment of cells with a specific src kinase family inhibitor, SU6656, or a broad spectrum kinase inhibitor, St638, did not prevent PMT from stimulating the covalent modification of Gα_q_ and Gα_i_, despite each kinase inhibitor being effective at blocking FAK phosphorylation. It is possible that the kinase inhibitors failed to completely block PMT-stimulated phosphorylation of G-proteins, due to their competitive nature and the enzymatic nature of PMT. However, this would suggest that deamidation by PMT results in the stable phosphorylation of these Gα subunits that is not reversed by the action of phosphatases, which is unlikely.

Deamidation and tyrosine phosphorylation of a Gα subunit would have a similar effect on the isoelectric point. The PMT-induced deamidation of in-vitro translated Gα_q_ and recombinant Gα_i-2_ was reported to cause an acidic pI shift of 0.05 and 0.07, respectively [14]. This compares with the acidic pI shift of approximately ∼0.15 for both Gα_q_ and Gα_i_ that we have observed. There are various possible interpretations of this apparent discrepancy. First, pI shifts are known to be variable and depend on the overall pI of a protein and its local context [34], and thus Gα_i_ expressed in *E. coli* may behave differently because of the absence of post-translational modifications. Alternatively, the PMT-induced modification in cells may differ from that observed following expression in *E. coli*.

PMT is reported not to activate G_11_, as PMT could not induce the activation of PLC in G_q_-deficient cells [12], and further analysis using Gα_q_/Gα_11_ chimeras also confirmed that PMT did not lead to G_11_-linked stimulation of PLC [35].We were therefore surprised that PMT stimulated the covalent modification of Gα_11_. Gα_q_ and Gα_11_ each contain Gln-209 that is functionally equivalent to Gln-205 in Gα_i-2_ and it would be unlikely that Gα_11_ could be deamidated and yet not activated by PMT, as the loss of the functional Gln would affect the GTPase activity of the G-protein. While this manuscript was in preparation, Kamitani *et al.* published evidence that an antibody against deamidated Gα subunits recognised Gα_11_ in PMT-treated mouse embryonic fibroblasts that were deficient in Gα_q/11_ but transfected to express Gα_11_ [36]. This result provides further evidence that G_11_ is also a substrate for PMT. In their experiments there was a small stimulation of PLC in cells expressing Gα_11_. All the other papers addressing this issue have used the same source of G_q/11_-deficient MEF cells, whereas our work uses Swiss 3T3 cells. Further investigation of these puzzling and partially contradictory results is required.

PMT treatment of cells led to new Gα_13_ isoforms at slightly higher (0.09–0.15) pI values. The PMT catalytic triad has high structural similarity to eukaryotic transglutaminases [14], and it is possible that PMT can also function as a transglutaminase, in a similar manner to the cytotoxic necrotizing factor (CNF) which was originally considered to be a deamidase, but was later found to cause transglutamination in cells [37]. Transglutaminases catalyse the acyl transfer between the γ-carboxyamide of a peptide bound glutamine (acyl donor) to a primary amine (acyl acceptor). When water functions as an acyl acceptor the result is glutamine deamidation [38]. The choice between deamidation and transglutamination is influenced by the environment of the targeted glutamine residue [39], [40]. As transglutamination would impart a positive charge to produce an alkaline shift, it is possible that PMT preferentially transglutaminates Gα_13_ in cells.

The removal of G-proteins from the membrane is a regulatory phenomenon that can follow prolonged G-protein activation [41]. The ADP-ribosylation of G_s_ by cholera toxin leads to its downregulation, although ADP-ribosylation of Gα_i_ by pertussis toxin does not result in its degradation [42]. We observed that prolonged treatment of cells with PMT caused the loss of Gα_i_, but not Gα_q_, from membranes prepared from Swiss 3T3 cells. Furthermore Gα_i_ could not be detected in the cytoplasm following prolonged PMT treatment. Orth *et al.* had suggested that overnight treatment of Swiss 3T3 cells with 1 nM PMT uncoupled Gα_i_ from its receptor, as the G_i_-linked agonist lysophosphatidic acid could not stimulate GTPγS binding to Gα_i_ in membranes derived from these cells [10]. The loss of G_i_ from the membrane that we observed over this time period would provide a more likely explanation for their observation. Furthermore, the site of the PMT-induced modification, Gln-205, is not thought to be linked to receptor interaction. A similar differential degradation has been observed with Rho proteins following modification by CNF [43].

We found that PMT could promote the binding of GTP to Gα_q/11_, whereas bombesin could not, which suggested that the action of PMT inhibits the GTPase activity of Gα_q/11_. PMT significantly inhibited the bombesin-mediated stimulation of steady-state GTPase activity in Swiss 3T3 membrane preparations. These results complement the demonstration that PMT inhibits the GTPase activity of *E. coli*-expressed Gα_i_ [10], [14]. Furthermore, pre-treatment of cells with cholera toxin and PMT resulted in a greater inhibition of GTPase activity, supporting the view that PMT does not affect G_s_.

In conclusion, our results demonstrate that treatment of Swiss 3T3 cells with PMT induces the irreversible modification of G-proteins belonging to the G_i_ and G_q_ families resulting in an acidic pI shift, which is consistent with the observation that PMT catalyses deamidation of recombinantly expressed G_i_ causing a similar shift in pI. We found that PMT inhibits the intrinsic GTPase activity of G_q_, which complements the finding that PMT-stimulated deamidation of Gα_i-2_ inhibits its GTPase activity. We showed that stimulation of cells with PMT results in the degradation of G_i_ which provides an explanation for the observation that PMT-treatment blocks G_i_ activation by a receptor agonist. The unexpected modification of Gα_11_ requires further investigation. We demonstrated that PMT treatment causes an alkaline pI shift in Gα_13_ and speculate that PMT might preferentially transglutaminate Gα_13_. Working with cells enables the PMT/G-protein interaction to be investigated in a more natural context than when working with recombinantly expressed proteins. However, the further interpretation of results is impeded by the near impossibility of purifying these low abundance proteins in a modified form from cell lines, and thus both in-vitro and in-vivo studies are required to unravel the complexity of the toxin/G-protein interactions.

## Supporting Information

Figure S1 **PMT does not induce the covalent modification of Gα_s_.** (**A**) Membrane proteins from Swiss 3T3 cells were separated by SDS PAGE and Western blotted with anti-Gα_s_ antibody. Membrane proteins from Swiss 3T3 cells left (**B**) untreated or (**C**) treated with 150 pM PMT for 4 h were separated by 2-D gel electrophoresis and Western blotted with anti-Gα_s_ antibody. Samples from at least 3 independent experiments were resolved with similar results.
(TIF)

Figure S2 **Kinase inhibitors do not block PMT induced modification of Gα_q/11_ or Gα_i_.** Swiss 3T3 cells were either not treated (Lane 1) or pre-treated (Lane 2) for 1 h with (**A**) SU6656 or (**B**) St638, then stimulated with 0.5 nM pervanadate for 5 min. The cells were lysed in SDS-buffer and proteins were resolved by SDS PAGE followed by Western blotting with an anti-phospho-FAK antibody. Three independent experiments gave similar results. Swiss 3T3 cells were (**C**, **D**, **G**, **H**) not treated or pre-treated with either (**E**, **I**) SU6656 or (**F**, **J**) St638 and then either treated with (**D**, **E**, **F**, **H**, **I**, **J**) 150 pM PMT or (**C**, **G**) not treated with PMT. Samples were resolved from 3 independent experiments with similar results. Membrane proteins were separated by 2-D gel electrophoresis and Western blotted with (**C–F**) anti-Gα_q/11_ antibody or (**G–J**) anti-Gα_i-1-3_ antibody. Samples were resolved from 2 independent experiments with similar results.
(TIF)

Figure S3 **Mutant PMT does not induces the covalent modifications of Gα_q_ or Gα_i_.** Membrane proteins from Swiss 3T3 cells (**A, C**) left untreated or (**B, D**) treated with 150 pM PMT^C1165S^ for 4 h, separated by 2-D gel electrophoresis and Western blotted with either (**A, B**) anti-Gα_q/11_ or (**C, D**) anti-Gα_i-1-3_ antibodies. Samples from 3 independent experiments were resolved with similar results.
(TIF)

Table S1 **Analysis of pI values of G_s_ family isoforms after treatment with PMT.** The samples were as described in the legend to Figure. S1 and the results are expressed as the mean ± standard error of the mean.
(DOC)
