# Supplementary figures and images for: Modification of Heterotrimeric G-Proteins in Swiss 3T3 Cells Stimulated with Pasteurella multocida Toxin
Source: PLoS One. 2012 Nov 5;7(11):e47188. doi: 10.1371/journal.pone.0047188 (PMC3489841; doi:10.1371/journal.pone.0047188)

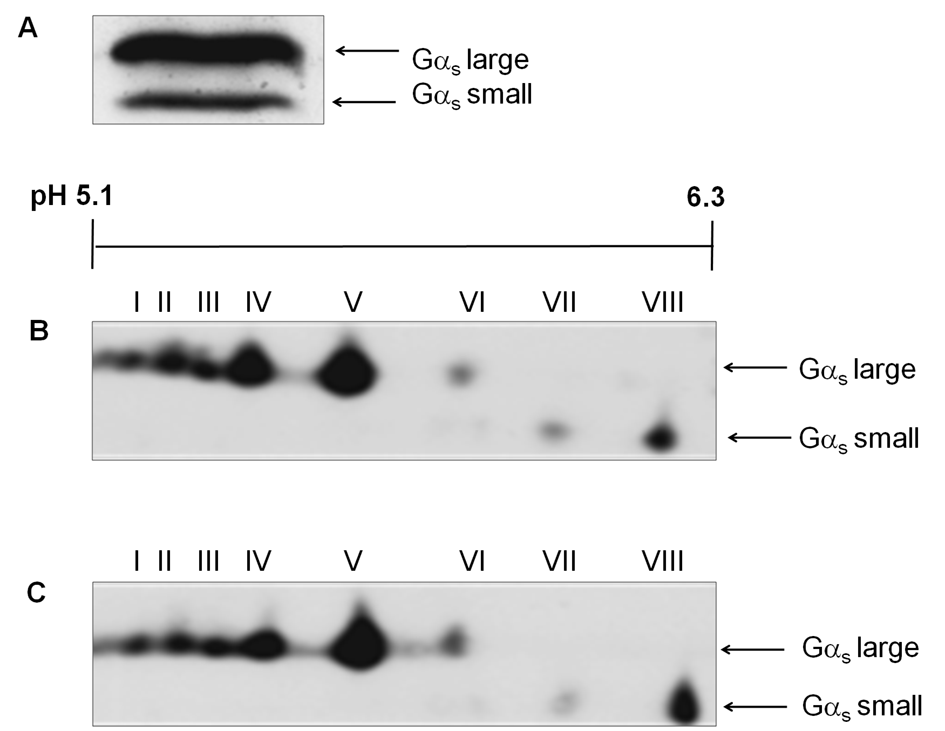

Supplement: Figure S1 — PMT does not induce the covalent modification of Gαs. (A) Membrane proteins from Swiss 3T3 cells were separated by SDS PAGE and Western blotted with anti-Gαs antibody. Membrane proteins from Swiss 3T3 cells left (B) untreated or (C) treated with 150 pM PMT for 4 h were separated by 2-D gel electrophoresis and Western blotted with anti-Gαs antibody. Samples from at least 3 independent experiments were resolved with similar results. (TIF) [file pone.0047188.s001.tif]

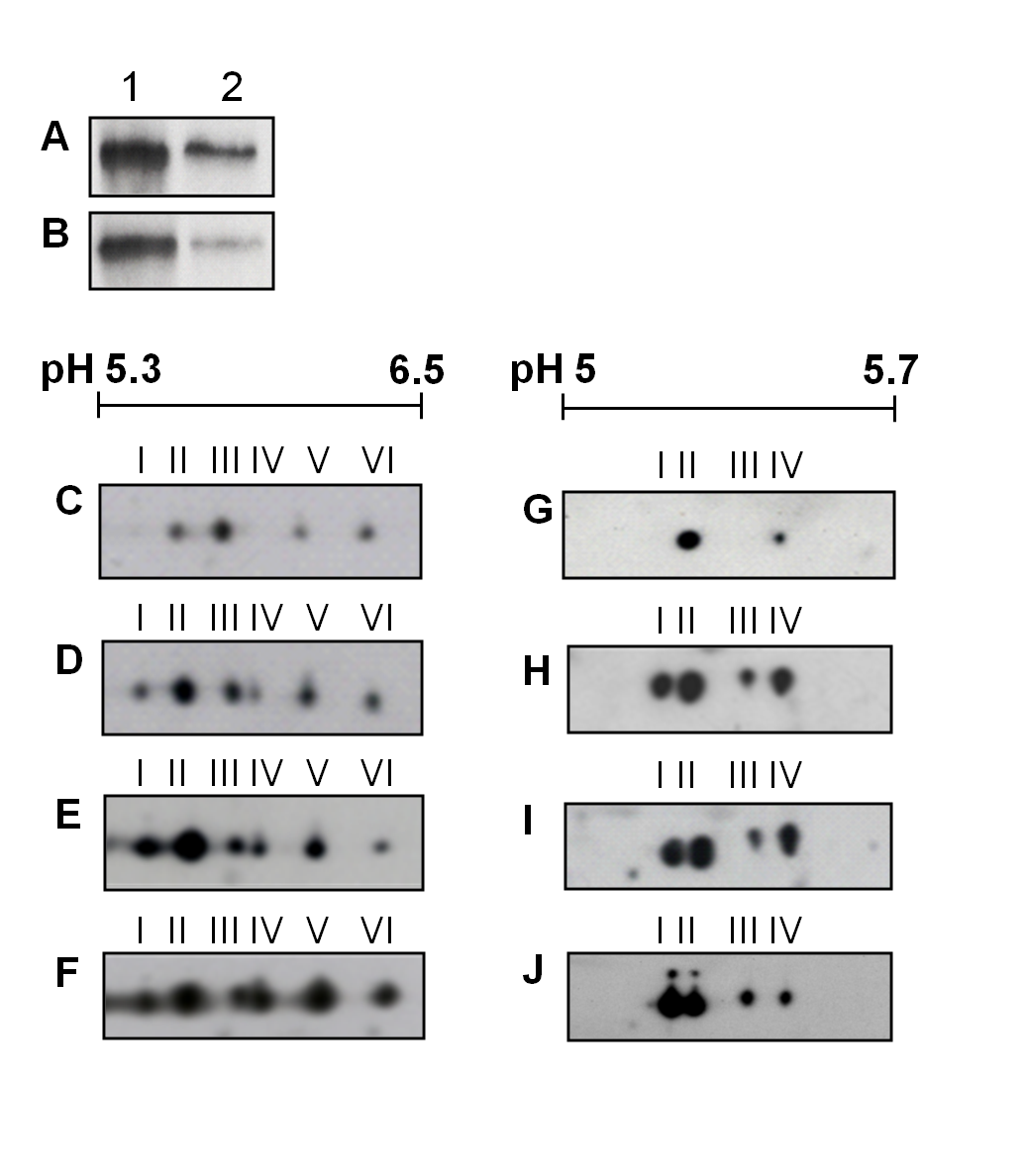

Supplement: Figure S2 — Kinase inhibitors do not block PMT induced modification of Gαq/11 or Gαi. Swiss 3T3 cells were either not treated (Lane 1) or pre-treated (Lane 2) for 1 h with (A) SU6656 or (B) St638, then stimulated with 0.5 nM pervanadate for 5 min. The cells were lysed in SDS-buffer and proteins were resolved by SDS PAGE followed by Western blotting with an anti-phospho-FAK antibody. Three independent experiments gave similar results. Swiss 3T3 cells were (C, D, G, H) not treated or pre-treated with either (E, I) SU6656 or (F, J) St638 and then either treated with (D, E, F, H, I, J) 150 pM PMT or (C, G) not treated with PMT. Samples were resolved from 3 independent experiments with similar results. Membrane proteins were separated by 2-D gel electrophoresis and Western blotted with (C–F) anti-Gαq/11 antibody or (G–J) anti-Gαi-1-3 antibody. Samples were resolved from 2 independent experiments with similar results. (TIF) [file pone.0047188.s002.tif]
