# Supplementary material for: Modification of Heterotrimeric G-Proteins in Swiss 3T3 Cells Stimulated with Pasteurella multocida Toxin
Source: PLoS One. 2012 Nov 5;7(11):e47188. doi: 10.1371/journal.pone.0047188 (PMC3489841; doi:10.1371/journal.pone.0047188)
Supplement: Table S1 — Analysis of pI values of Gs family isoforms after treatment with PMT. The samples were as described in the legend to Figure. S1 and the results are expressed as the mean ± standard error of the mean. (DOC) [file pone.0047188.s004.doc]

**Table S1. Analysis of pI values of G_s_ family isoforms after treatment with PMT**.

The samples were as described in the legend to Figure. S1 and the results are expressed as the mean ± standard error of the mean.

|  | **Control** | **PMT-treated** |
| --- | --- | --- |
| **Isoform** | **Gα_s_** | **Gα_s_** |
| **s-I** | 5.45 ± 0.02 | 5.40 ± 0.03 |
| **s-II** | 5.48 ± 0.03 | 5.48 ± 0.01 |
| **s-III** | 5.55 ± 0.03 | 5.53 ± 0.01 |
| **s-IV** | 5.60 ± 0.01 | 5.61 ± 0.01 |
| **s-V** | 5.77 ± 0.03 | 5.77 ± 0.03 |
| **s-VI** | 5.94 ± 0.01 | 5.91 ± 0.03 |
| **s-VII** | 6.12 ± 0.02 | 6.10 ± 0.01 |
